# Tuning in: How Hearing Loss and Assistive Devices Reshape Musical Quality of Life

**DOI:** 10.3390/audiolres16020054

**Published:** 2026-04-02

**Authors:** Felicity Bleckly, Emilie Francis-Auton, Frances Rapport, Robyn Clay-Williams, Chi Yhun Lo

**Affiliations:** 1 Australian Institute of Health Innovation, Macquarie University, Sydney, NSW 2109, Australia; 2 Department of Linguistics, Macquarie University, Sydney, NSW 2109, Australia; 3 Performance and Expertise Research Centre, Macquarie University, Sydney, NSW 2109, Australia

**Keywords:** cochlear implant, hearing aid, music-related quality of life, musician, non-musician

## Abstract

Background/Objectives: Hearing loss, coupled with the configurations of hearing devices, adds to the complexity of understanding the subjective and personal implications of losing musical fidelity. Hearing music through assistive listening devices significantly impacts music perception and enjoyment, yet research examining music-related quality of life for late-deafened adults is limited. This study aimed to capture late-deafened adults’ experiences related to music and quality of life. Methods: The study administered a cross-sectional survey designed around three established questionnaires: Cochlear Implant Quality of Life, Goldsmiths Musical Sophistication Index, and Music Related Quality of Life. It was completed by 116 late-deafened adults (mean age 65.4 years, with an average of 23.1 years of hearing loss). It was hypothesised that the use of different hearing devices would impact music importance, engagement, enjoyment, and related quality of life in disparate ways. To determine if and how quality of life differed between hearing device users, statistical analyses were stratified across a subgroup of 75 participants with bilateral hearing aids (n = 33; musicians n = 18, and non-musicians n = 15), bilateral cochlear implants (n = 21; musicians n = 5, and non-musicians n = 16), and bimodal configurations (n = 21) musicians n = 8, and non-musicians n = 13). Results: Music remained important for most participants (n = 55, 73%) despite hearing loss. However, regardless of music being valued, only 36 (48%) participants enjoyed music “Always” or “Most of the Time”, while 17 (23%) “Rarely” or “Never” enjoyed it. Bilateral hearing aid users reported the highest, and bilateral cochlear implant users the lowest quality-of-life scores. These effects extended to participation in real-world musical activities: hearing aid users attended more live music events, while bilateral cochlear implant users experienced the greatest reduction in musical activities compared to other hearing device users. Conclusions: Musical quality of life is fundamentally about music enjoyment and engagement and how late-deafened adults integrate music into their everyday life. Hearing loss and hearing devices create a profound disconnect between the capacity to enjoy and engage with music. Musicianship did not guarantee better musical enjoyment or engagement. However, musicians demonstrated greater perseverance when enjoyment was limited, in the hope of improvement. Understanding this allows clinicians to develop effective rehabilitation strategies tailored to different hearing devices and musicianship abilities and set realistic expectations.

## 1. Introduction

Hearing loss (HL) disrupts life expectations [1] for late-deafened adults (LDAs), people who once had typical hearing. Self-esteem may be challenged, with the constant need to re-evaluate what is considered “normal” while developing a new identity [1,2,3]. Approximately 466 million people worldwide report some degree of loss, with LDAs representing the largest proportion [4]. In Australia, this permanent sensory disability affects two in five people over the age of 65, and with an ageing population, this number will rise in the future [4].

### 1.1. Music and Quality of Life

Music is part of our daily existence, a cornerstone in every culture, an external sound shaping internal experience in meaningful ways [5,6]. It entertains, provides enjoyment, conveys ideas [7], and awakens memories. Music reflects social traditions, promotes social bonding [8], and can become a surrogate friend in times of loneliness [9,10], playing a vital role in health outcomes and quality of life (QoL) [11,12]. 

### 1.2. Hearing Device Limitations and Music Perception

Modern hearing aids (HAs) and cochlear implants (CIs) have had remarkable success in restoring speech perception, thus improving communication [13,14]. However, speech has a relatively small frequency range. Music is far more complex and includes competing sound streams through harmony, timbre, rhythm, instruments, or voice. HA technology amplifies sound but cannot activate dead regions in the cochlea [15,16,17,18]. This generally means that many of the frequency components in music will not be perceived. In contrast, CIs directly stimulate the cochlea and, often in a somewhat limited fashion, replicate missing frequencies. They provide temporal envelope cues, which are effective at restoring speech perception, but they cannot provide sufficient spectral information for fine frequency discrimination and music fidelity [19,20,21]. Thus, reproducing the acoustic complexities of music remains beyond the processing capabilities of current technology [22,23]. These technological limitations create a fundamental incongruity between LDAs’ continued love of music and the sound delivered through hearing devices. This mismatch between expectation and perception can impact enjoyment [24], often leading to decreased musical engagement. This may reduce happiness [25], and may be one of the more profound consequences of HL [26,27].

### 1.3. Consequences of HL on Music

One consequence of HL is that listening to new music may not be rewarding and may be frustrating [5,28]. Music enjoyment relies on musical “entrainment” (cognitive processing based on previous music experience) to build mental models of pattern recognition in music, generating anticipatory engagement and expectations of reward [13,29,30]. When auditory signals are impoverished, pattern extraction becomes effortful with limited pattern consolidation. Therefore, new musical experiences take cognitive energy and effortful listening [13,31]. No memories are laid down, and the cognitive–emotional loop that makes music meaningful is lost [13].

### 1.4. Conceptualising Musical Experiences and QoL

For the purpose of this study, musical QoL was assessed across multiple dimensions, recognising the complexity and interaction between music enjoyment, active engagement, perception of music, and overall importance of music. Music enjoyment includes the short-term hedonistic reward that comes from creating or listening to music, the personal emotional response, and the triggering of memories [32]. Active engagement recognises that music is not an object but an activity [33]—an important distinction because active participation is a significant predictor of general and musical QoL [22,34,35,36]. Perception relates to how well participants perceive the music pitch, melody, harmony, or timbre, including their assessment of it sounding the way they expect or remember. Musical sophistication is a combination of musical background, training, engagement, perceptual abilities, and the importance of music in participants’ lives. The importance of music, particularly for musicians, is a subjective and personal value [37]. Studies in CI populations found that musical sophistication correlates with music perception [37,38], suggesting that perceptual limitations may constrain musical sophistication. For LDAs, changes in perception present barriers to maintaining musical sophistication, with reported significant decreases in listening time compared to before HL [24,39], limiting music exposure essential for sustained engagement. Musical QoL reflects the overall satisfaction [38] with music experience [39] and hearing device use [40,41].

It is reported that LDAs spend significantly less time listening to music compared to before their HL and considerably less than typical hearing peers [26,42]. The degraded signal from hearing devices impairs accurate pitch discrimination, making it harder to identify melodies and perceive timbre, which frequently leads to limiting music exposure. 

### 1.5. Research Gap: Beyond Perception to Lived Experience

For people with typical hearing, music is often classified as a purely auditory experience [43], supporting the expectation that hearing devices return access to and enjoyment of music [44,45]. A recent systematic literature review [46] reinforced this point, finding the focus for music research, HL, and hearing devices is based around audibility—music perception—rather than the enjoyment and importance of music. Of 131 studies reviewed, 91 focused on CI users’ music perception, testing accurate identification of musical components, usually pitch, melody, timbre, and/or rhythm, outcomes of which were typically poorer compared to hearing peers. Nevertheless, as Akbulut et al. [47] remarked, there is only a weak correlation between music perception and music enjoyment. Focusing on perception alone misses the personal importance and meaning of music, subjective dimensions which may be more relevant to the user experience and more critical for psychosocial wellbeing and QoL than accurate perception.

### 1.6. Study Aims

In the last decade, the research landscape of HL has changed significantly, with greater incorporation of the stakeholder perspective to drive research questions leading to improved design of HAs or CIs [24,48]. However, research into subjective musical experiences for LDAs has been sparse [49], highlighting a major gap in understanding the personal issues surrounding music. The primary objective of the current survey was to characterise broad patterns of LDAs’ experiences and the interaction between HL, hearing devices, and music. The research aimed to answer the question: What are the implications of HL and hearing devices for LDAs’ relationship with music, and how does this affect their QoL and wellbeing?

### 1.7. Research Hypotheses

To address the gap, this project explored music importance, enjoyment, active engagement, and perception to understand which musical facets have the most impact and how each relates to the others to impact QoL. To answer the research question, we assumed the following hypotheses:

**Hypothesis 1** **(H1).** *Better musicality, as measured by its importance, enjoyment, engagement, and perception, would be positively correlated with QoL.*

(EQ1) *We explored which of these factors (importance, enjoyment, engagement, and perception) contributes most to QoL.*

**Hypothesis 2** **(H2).** *Because of perceptual difficulties, we predict that music sophistication for LDAs would be lower than that of typical hearing people, and this will negatively impact QoL.*

**Hypothesis 3** **(H3).** *Due to perceptual difficulties, we hypothesised that participation and engagement in musical activities would be lower for LDAs than in the general population.*

We expected participants who self-identified as musicians to show stronger music-related effects compared to non-musicians, and that hearing device type would also play a role. 

## 2. Materials and Methods

**Figure 1:**
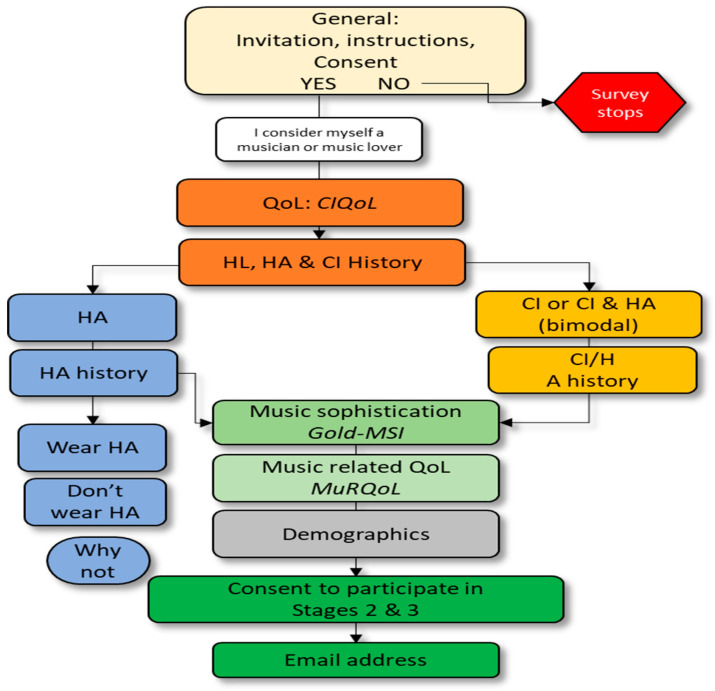
Demographic, music sophistication, and QoL survey flowchart. The colors indicate different sections of the survey.

The study used a cross-sectional design (Figure 1) to capture responses at one point in time from subgroups of LDAs, considering the potentially confounding issues of musicianship, different degrees of HL, and diverse hearing device configurations.

The survey incorporated three validated questionnaires, which allowed comparison of participants’ music-related QoL with normative findings of typical hearing people. The first questionnaire, Cochlear Implant Quality of Life (CIQoL) [50], measured general QoL. It included 35 questions over six domains: communication, emotional, entertainment/music, environmental, listening effort, and social connections. It was tested for reliability and reported as psychometrically sound by MacRackan et al. [50,51] and used by other researchers. Laplante-Lévesque [52] stated CIQoL was valid for people with HL before receiving a CI, and both Yang [53], and Mitton et al. [54], used the CIQoL assessment preoperatively to allow a comparison of QoL before and after CI surgery.

The second questionnaire, Goldsmith’s Musical Sophistication Index (Gold-MSI) [55], focused on assessing music sophistication in the general population but did not specifically address HL. Gold-MSI was created in collaboration with the Universities of London and Vienna, validated in studies by the creators [56], used in several other studies in the general population [57,58], and used to assess musical sophistication for those with HL [59,60]. It measured attitudes, behaviours, music skills, and experiences across domains (active engagement, perceptual abilities, musical training, singing abilities, emotions) to create a global music sophistication score. The global score is a separate construct based on 18 selected variables from within each domain. These included recognising music, active engagement, enjoying new music, or whether someone considered themselves a musician, attended music events, and listened for minutes per day.

The third questionnaire, music-related quality of life (MuRQoL) [24], included 18 questions, enabling correlation of the perception of music with the importance of music. It has been validated in several studies covering CI recipients [24,35,61,62]. According to the MuRQoL authors, five questions are novel compared to other music questionnaires. Three of these were subjective variables: (1) ability to judge the quality of music, (2) confidence that they hear music the way other people do, and (3) ability to hear the emotion in the music. The other two variables were related to perception or context: (4) ability to recognise words in music and (5) ability to listen to music in the car. The outcomes from the questionnaire enable the importance music holds for a participant to be significantly correlated with their perception, which impacts their ability to enjoy it.

Additional questions were added for this study to enable the identification of LDAs by the hearing device they used. Demographic questions were placed last to gain trust before participants could optionally proffer personal details, after which they could “opt in” to participate in additional stages of the project by providing an email address. Data on additional questions were not included in the statistical analysis except to stratify participants by musicianship and hearing device.

### 2.1. Participants

To understand the influence of any personal characteristics of the researched population, additional data captured included demographics (age, gender, education, employment, country), plus HL history (age of HL onset, slow or sudden progression, symmetry/asymmetry of HL between ears, and duration of HL) and assistive hearing devices used. The research targeted only LDAs, as we sought to quantify the experience of those who once knew hearing privilege [63,64] and enjoyed music primarily through hearing, even if they also experienced audiation, physical and emotional responses to, and recognised meanings in music [65]. Study exclusion criteria were those who experienced pre-lingual HL and those who identified with Deaf culture. Deaf communities have a strong, vibrant culture with a rich history, communicating using unique sign languages [66]. However, their music experiences mostly rely on visual and tactile stimulation, which are conceptually different from those of LDAs who once enjoyed the sound of melodies, harmonies, and musical instruments [67,68].

### 2.2. Recruitment Strategy

LDAs may be marginalised and form a small heterogeneous sub-population within mainstream culture, often hidden and not easily identifiable. Therefore, using social media as the first contact point was selected as a purposive–convenience sampling method [69]. Of the many social media platforms available in English, Facebook was chosen because it is the platform most likely to reach older adults [70,71]. A search of Facebook revealed more than 133 groups which involved HL or hearing devices, but not all included LDAs. After examination, 13 of these were deemed appropriate for this research because members were primarily LDAs with HL and/or HAs (n = 4 groups), CI recipients (n = 6 groups), or musicians with HL, HAs, or CIs (n = 3 groups) (Appendix A, Table A1). In addition to the Facebook groups, HL professionals, Cochlear Implants South Australia [72], and Better Hearing Australia [73] emailed the invitation to patients, plus the Australian Hearing Hub [74] placed the invitation on their research website (Appendix A, Table A2).

Participants were also asked to send the survey link to people they may know who fit the participation criteria. Snowball sampling [75] is a non-probability technique frequently used in research with marginalised populations who are vulnerable and/or hard to reach, and this method had the potential to increase the diversity of participants [76]. Through opportunistic sampling [75], people who met the inclusion criteria and were known to the researcher were contacted. This was particularly important to increase response from the hardest-to-reach group (HL or HA non-musicians). Given recruitment challenges, as has been seen in other studies in hidden populations [77], it was deemed a minimum of 100 responses [78] would represent a sizeable portion of accessible participants. The study was open between late September 2023 and April 2024, and no incentives were offered for participation.

### 2.3. Data Collection

The survey was created in LimeSurvey 2021 [79] with invitations to participate posted to 13 online Facebook HL, HA, CI, and HL musician groups. Participants needed to acknowledge informed consent and agreement to participate before they could commence answering questions.

### 2.4. Analysis Tools

SPSS 27.0 2020 [80] was utilised for all statistical analyses. Sun et al. [81] suggest generative AI has gained increasing recognition in recent years for extracting patterns and conclusions for those who lack statistical training. In this current study, AI tool (Claude 2025) [82] were used to assist with understanding statistical analysis and the phraseology of reporting statistics. Nevertheless, to guard against any interpretation errors, all analyses were conducted by the authors and validated against raw data outputs. A multiple linear regression examined which music-related factors predicted QoL. The significance level for statistical tests was set at α = 0.05, and Bonferroni corrections were included for multiple analyses. All significance tests were conducted using two-tailed tests where applicable.

### 2.5. Data Preparation

Data preparation was completed in accordance with published procedures for the CIQoL [83], Gold-MSI [84], and MuRQoL [85] questionnaires. Likert scales were converted to numerical scores, reversing responses for specific questions and calculating overall scores. The identification of subgroups (Table 1) limited the statistical analysis to those who completed the survey. Cleaned data were imported to SPSS [80], where all non-categorical variables were tested for normality using Q-Q plots or histograms, and all outliers were examined. Sensitivity analysis was conducted for all variables, with and without outliers (10.7% of the subgroup), to assess the robustness of findings. Most outliers were identified in the CIQoL social domain; however, removing outliers had an insignificant impact on the mean (M = 53.67 versus M = 53.66), although this did slightly reduce variability. Therefore, to maximise statistical power, all outliers were retained based on the following:

- Many questions were subjective, so there were no right or wrong answers.
- Individual characteristics and experiences of LDAs with HL/hearing devices vary significantly.
- Eliminating outliers may silence participants who have the most challenges.
- Where applicable, due to the skewness of outliers, a median was calculated rather than a mean (e.g., listening hours and music event attendance).

**Table 1:** Summary of subgroup sizes by hearing device worn and musicianship: demographic characteristics.

	Bilateral HAs	Bilateral CIs	Bimodal	Total
Musicians	18	5	8	31
Non-Musicians	15	16	13	44
Total	33	21	21	75

### 2.6. Identifying Subgroups—(1) Hearing Device Type and (2) Musicians or Non-Musicians

Frequencies for all survey responses were cross-tabulated by hearing device, resulting in seven different hearing device groups. Four of the subgroups, no hearing device (n = 2), unilateral HA (n = 9), unilateral CI (n = 14), and hybrid CI (n = 6) (CI and HA in the same ear), were excluded from analyses due to small sample sizes. Table 1 shows the final selection, based on musicianship and hearing device, of participant groups which were statistically analysed.

**Figure 2:**
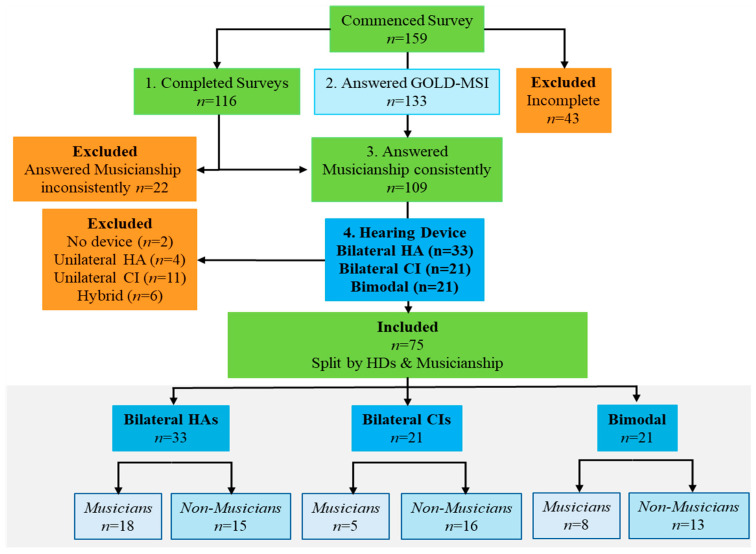
CONSORT diagram showing groups included in statistical analysis. Orange = excluded participants, Green = Survey participants, Blue = frequency analysis by hearing devices and musicianship.

Musicianship was based on self-identification as a musician or non-musician and was not judged on any formal musical tests [26,27]. Musicians may be amateur or professional, creating music in any form, including as singers, conductors, instrumentalists, DJs, or other roles [56]. Non-musicians may have had formal training but self-identify only as music consumers, avid listeners, or those who attend musical performances. Musicianship was also correlated with the number of years participants practised (>6 years n = 31) and participated in training (4–6 years n = 2, >7 years n = 24) [86]. The training of self-identified non-musicians was considerably less than for musicians (4–6 years n = 7, >7 years n = 1). Nevertheless, these participants were still analysed as non-musicians. One of the early questions was that participants were asked to select “Yes” or “No” in response to the statement “I consider myself to be a musician”. At this early stage in the survey, 74 participants identified as musicians and 85 as non-musicians. This question was revisited in the Gold-MSI questionnaire section and while 133 participants answered, only 109 answered consistently for both “musician” questions—musicians (n = 43) and non-musicians (n = 66). However, not all 109 completed the survey or used the hearing devices identified, which further reduced the final sample size for statistical analysis to 75 (Table 1). The group selection process for analysis is identified in the CONSORT diagram (Figure 2).

## 3. Results

The survey took an average of 25 min (between 11 and 62 min) to complete, with 159 participants commencing and 116 (73%) completing all questions. Data were imported from LimeSurvey [79] into Excel [87]. At this point, preparation of data for analysis was undertaken as per instructions from CIQoL [83], the Gold-MSI online tool [55], and MuRQoL [85]. Descriptive statistics were completed for all variables to calculate frequency, mean, range, and standard deviation. There were no missing data in CIQoL, Gold-MSI, or MuRQoL responses. There were four respondents who did not answer one of two questions (duration of hearing device use, n = 4; occupation, n = 1). However, these questions were not used in statistical analysis.

### 3.1. Demographics

Participants were heterogeneous, and there was a non-normal distribution for most demographics (Appendix A, Table A3). The majority were aged between 60 and 80 years (M = 65.4, SD = 11.8), with 48 identifying as female (M = 62.63 years, SD = 12.9) and 27 identifying as male (M = 70.4 years, SD = 7.3). More LDAs were retired (female n = 29, male n = 19) than in employment (female n = 15, male n = 8). The participant pool was well educated, with 56% (n = 42) holding university degrees. However, the proportion with high school (n = 11) and college qualifications (n = 7) plus trade certificates (n = 1) suggests participants came from a range of educational backgrounds. LDAs had substantial experience with hearing device use. However, there was a difference between the length of HA use (M~13 years) and that of using a CI (M~4–6 years) (Appendix A, Table A4).

### 3.2. Enjoyment, Engagement, and Perception Would Be Positively Associated with QoL (H1, EQ1)

A multiple linear regression examined which music-related factors (enjoyment, engagement, importance, or perception) predicted QoL (CIQoL). The overall model was significant, F(4, 70) = 4.865 and *p* = 0.002, explaining 17.3% of variance in QoL (adjusted R^2^ = 0.173). Collinearity diagnostics were conducted to assess multicollinearity among predictors (i.e., no overlap between variables). Acceptable levels of multicollinearity were found, indicating each predictor contributed unique variance to the model with variance inflation factors (VIF) below 3 (range: 1.39–2.64).

Greater enjoyment of music was the strongest contributor to QoL (β = 0.467, *p* < 0.001), which significantly improved QoL. Active engagement with music was also a significant aspect of QoL (β = −0.350, *p* = 0.046). However, when controlling for music enjoyment and perception, active engagement showed a negative relationship, suggesting a suppression effect. This means active music engagement (playing instruments, listening, singing) by itself does not relate to QoL (r = 0.023).

Self-rated importance of music (β = 0.149, *p* = 0.248) and music perceptual abilities (β = 0.106, *p* = 0.526) did not individually predict QoL when accounting for other music-related factors. These relationships were investigated further through subgroups: (1) musicianship and (2) hearing devices (Table 2).

#### 3.2.1. Musicians Versus Non-Musicians

It was expected musicians would consider music of higher importance than non-musicians. All musicians (100%) rated music as always important, but this did not translate to enjoyment. Only 39% of musicians always enjoyed it, with two musicians stating rarely, and another never enjoying it. In contrast, non-musicians showed more variation in both importance and enjoyment; 55% rated music as always important, and 33.3% enjoyed it most of the time. It should be noted that the linear regression was unreliable for musicians because music was important 100% of the time (Table 2). 

#### 3.2.2. Hearing Device Configurations

It was found that the enjoyment of music varied, depending on type of hearing device; 90% of bilateral HA users reported music was always important to them. HA users enjoyed music higher than users of any CI configuration with only 66% of bilateral CI users and 62% of bimodal users saying music was always important to them. The mismatch between importance and enjoyment was most pronounced in bilateral CI users. Of those who reported that music was always important, only 29% were able to enjoy it. Table 3 shows that hearing device configuration appears to impact musical enjoyment.

Linear regression analysis suggests that, depending on the hearing device type, music-related factors predict QoL differently. Music enjoyment (β = 0.519, *p* = 0.002) for HA users had the strongest positive effect, with perception having marginal significance (*p* = 0.057). For bilateral CI users, none of either the enjoyment, importance, engagement, nor perception were significant, although perception showed a trend (*p* = 0.076). For bimodal users, linear regression was significant (*p* = 0.004), although none of the individual predictors reached full significance. 

### 3.3. Exploring Music Importance, Enjoyment, Engagement, Perception, and QoL (H1, EQ1)

If music was deemed important, it was expected participants would pursue musical activities even if enjoyment was missing, in the hope of improvement, and this would be stronger for musicians than non-musicians. Two questions at the beginning of the survey asked participants to rate the importance of music and how much they enjoyed music. Descriptive statistics revealed that for 73.3% (n = 55) of participants, music was always important, and none indicated that music was never important. The enjoyment of music was more varied, with only 22.7% (n = 17) always enjoying it, a further 34.7% (n = 26) enjoying it most of the time, and 20% (n = 15) sometimes enjoying music. In contrast, 14.7% (n = 11) rarely enjoyed music, and 8% (n = 6) could never enjoy it. A Spearman’s rho correlation revealed there was a significant positive linear correlation between the importance and enjoyment of music (rs = 0.289, *p* = 0.012).

To test the connection between music perception and the importance of music, the MuRQoL questionnaire scores for participants’ perception (M = 50.45) versus the importance of music (M = 65.27) were analysed. Pearson’s correlation showed no significant linear relationship between perception and importance scores (r = 0.267, *p* = 0.20).

Based on the alignment between importance and perception levels, participants’ responses were reclassified into four QoL categories (Table 4). The continuous correlation was not significant, but after categorisation of data into the QoL categories, chi-square analysis revealed a significant association (χ^2^ (1, N = 75) = 5.36, *p* = 0.021) between perception and importance. Most participants’ scores (n = 36, 48%) were classified as strong positive QoL (high importance + high perception), where music is both valued and well-perceived. Nearly one-third of scores (n = 24, 32%) were classified as strong negative QoL (high importance + low perception), representing a mismatch where music is highly valued but poorly perceived. The rest of the participants’ scores, based on the relationship between importance perception levels, were classified as weak negative QoL (n = 11, 14%; low importance + low perception) or weak-positive QoL (n = 4, 5%; low importance + high perception).

#### 3.3.1. Musicians Versus Non-Musicians

Statistical analysis investigated whether musicianship impacted LDAs’ music perception–importance relationship (Table 5). As Levene’s test (F = 20.23, *p* < 0.001) indicated equal variances were violated, a one-way Welch ANOVA revealed a significant difference in the QoL influence between musicians (M = 3.71, SD = 0.53) and non-musicians (M = 2.73, SD = 1.15). Even with a Welch correction (F(1, 64.41) = 4.75, *p* < 0.001, η^2^ = 0.21), 77% of musicians were significantly more likely to report positive impacts (weak positive n = 1, strong positive n = 23) compared to 36% of non-musicians (weak positive n = 3, strong positive n = 13).

#### 3.3.2. Hearing Devices

**Figure 3:**
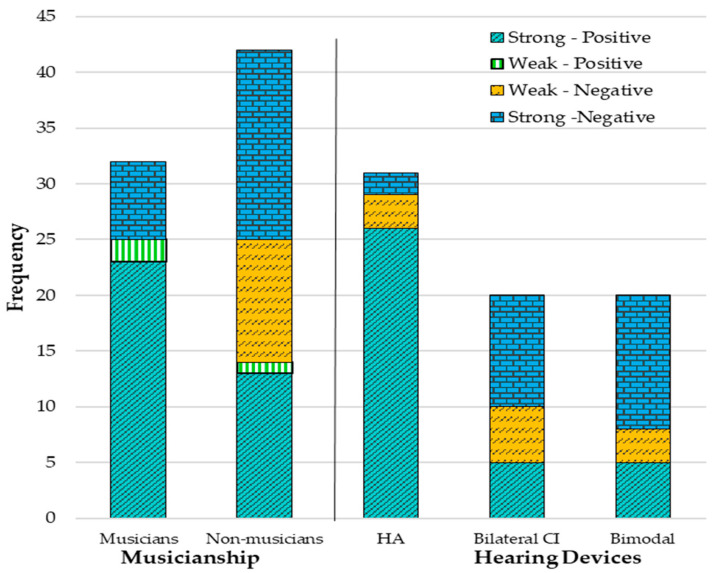
MuRQoL perception vs. importance of music—musicianship and hearing devices.

The same analysis used for musicians/non-musicians was used to investigate differences in MuRQoL scores based on hearing devices used (Table 5). A one-way ANOVA revealed statistically significant differences in MuRQoL overall perception–importance scores between device types. Post hoc Games–Howell tests identified that bilateral HA users scored 15 points higher (*p* = 0.009) than bilateral CI users and 13 points higher than bimodal wearers (*p* = 0.005). Bilateral CI and bimodal users showed no significant difference. The bar chart in Figure 3 illustrates the positive and negative impacts of musicianship and hearing devices on QoL.

Two-way ANOVA examined the effects of hearing device type and musicianship on MuRQoL components. Perception was significantly associated with hearing device type (*p* < 0.001) and musicianship (*p* = 0.002) (Table 6), but there was no significant interaction (*p* = 0.408). Tukey post hoc analysis revealed that HA users scored significantly higher (M = 61.76) than both bilateral CI users (M = 40.97) and bimodal users (M = 42.16). Bilateral CI and bimodal users did not differ significantly (*p* = 0.967). Importance of music revealed statistically significant differences between musicians and non-musicians (*p* < 0.001). However, hearing devices did not affect importance (*p* = 0.557), and there was no significant interaction (*p* = 0.063). Nevertheless, musicians (M = 76.09) demonstrated substantially higher importance than non-musicians (M = 57.65) regardless of hearing device. Overall MuRQoL scores demonstrated that both hearing device type and musicianship independently were associated with musical QoL, with no significant interaction. HA users reported significantly higher musical QoL (M = 65.80) compared to both bilateral CI users (M = 50.91) and bimodal users (M = 52.35). Musicians reported higher overall musical QoL (M = 68.17) compared to non-musicians (M = 50.60) across all device types (Appendix A, Table A5).

### 3.4. LDAs’ QoL—Musicians Versus Non-Musicians and Hearing Devices

**Figure 4:**
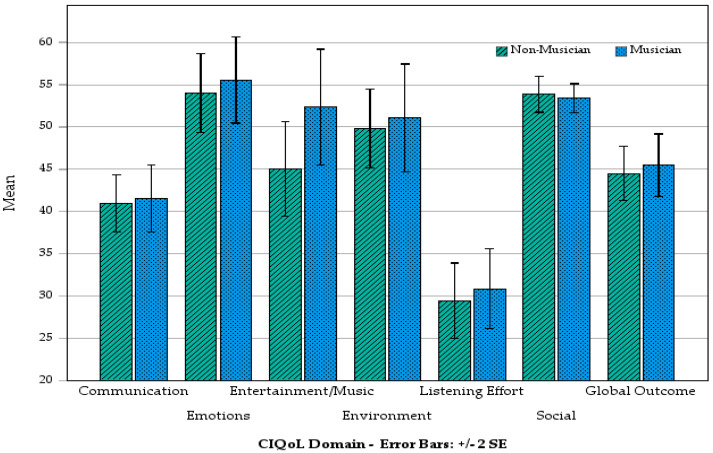
CIQoL domain outcomes by musician/non-musician.

**Figure 5:**
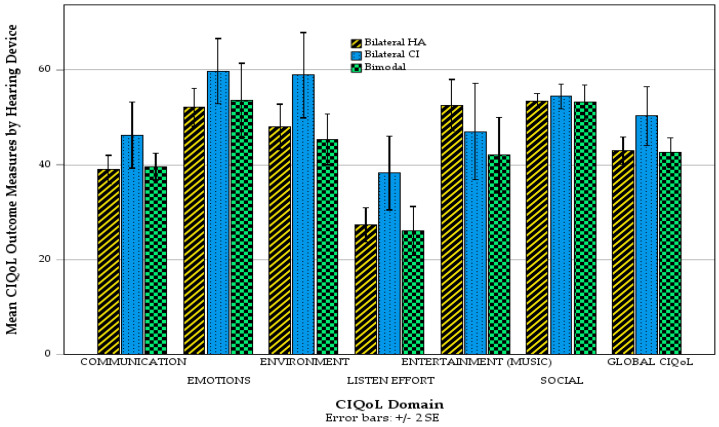
Mean CIQoL domain outcomes by hearing device.

To investigate QoL outcomes in the current study, independent samples *t*-tests compared musicians and non-musicians and hearing devices individually across all seven CIQoL domains with Bonferroni corrections applied (Figure 4). No statistically significant differences were found for any domain. One-way ANOVAs with Games–Howell post hoc tests (which do not assume equal variances) compared outcomes for all CIQoL domains individually between the three device groups (bilateral HA, bilateral CI, and bimodal). No pairwise comparison revealed any statistical significance after Bonferroni correction. Figure 5 presents the mean CIQoL domain scores of those with different hearing devices.

### 3.5. LDAs’ Music Sophistication Compared to Typical Hearing People (H2)

Gold-MSI normative data for music sophistication of typical hearing people in the general population was obtained for each of the six Gold-MSI domains [56]. One-sample *t*-tests compared Gold-MSI (n = 147,633) scores to LDAs scores (n = 75), and five of the six domain scores (active engagement, perceptual abilities, singing abilities, emotions, general musical sophistication) were significantly lower than the norm, after Bonferroni correction (six comparisons). A comparison of music sophistication between LDA adults and the general population norms is presented in Appendix A, Table A6 and Table A7. The training domain for LDAs (*p* = 0.575) did not significantly differ from the norms. This was explored further through subgroups, musicianship, and hearing devices.

#### 3.5.1. Musicians Versus Non-Musicians Versus ALL Participants

**Figure 6:**
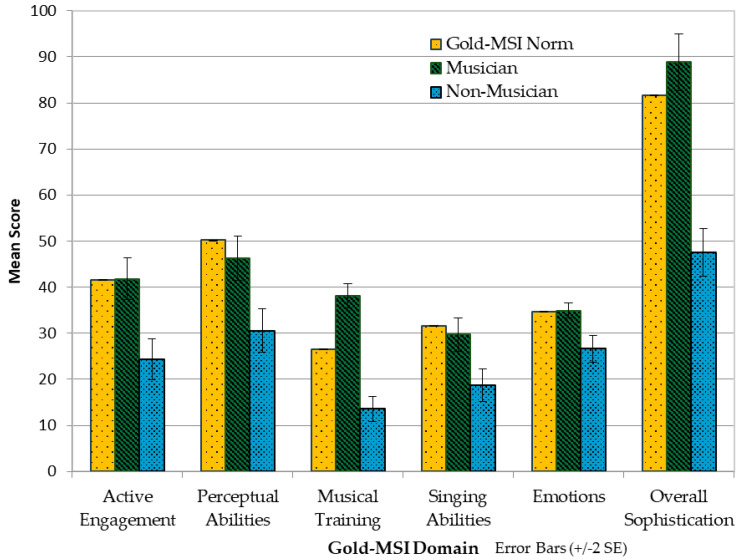
Gold-MSI domains by musicians and non-musicians.

As shown in the table in Appendix A, Table A6, LDAs in the current study were stratified by musicians (n = 31) and non-musicians (n = 44), and scores were compared to the Gold-MSI normative using independent samples *t*-tests over each of the Gold-MSI domains. There were no statistically significant differences between the LDA musicians and the normative data from the general population for all domains except in musical training, with the LDA musicians’ scores significantly higher (LDA = 38.13; Gold-MSI norms = 26.52, *p* < 0.001). When comparing the scores between the LDA non-musicians and norms, all domains were significantly lower, suggesting musicianship may mitigate some of the differences. Figure 6 compares the Gold-MSI norms with musicians’ and non-musicians’ scores.

#### 3.5.2. Hearing Devices

**Figure 7:**
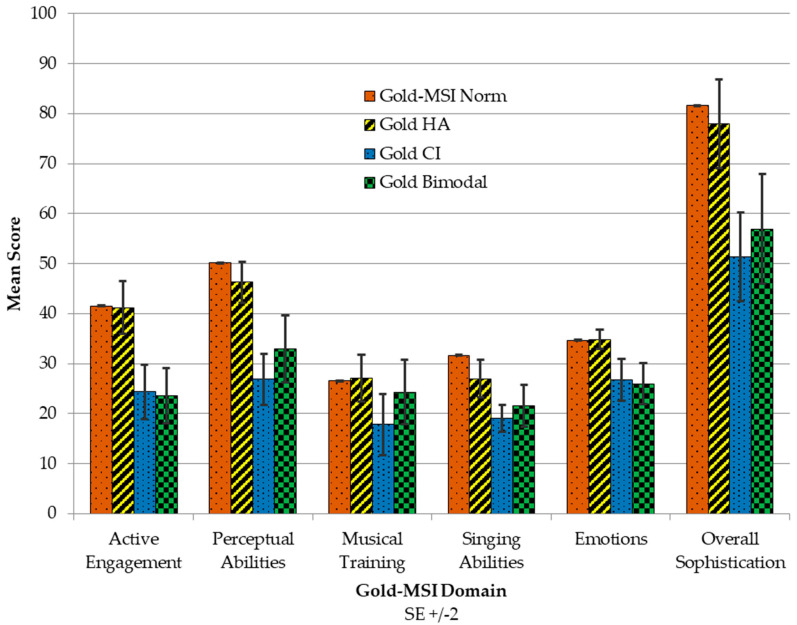
Gold-MSI domain outcomes by HA, bilateral CI, and bimodal.

It was hypothesised that different hearing technologies would impact LDAs’ musical experiences in different ways. Appendix A, Table A7 shows the comparison of scores between LDAs’ different hearing devices and the norms. HA users scored close to the Gold-MSI norms. However, most of the scores for bilateral CI and bimodal devices were significantly lower than the Gold-MSI norm. The exception was musical training. Comparing hearing device users’ scores with the Gold-MSI normative provided an indication of the impact hearing devices have on music, regardless of musicianship status. As depicted in Figure 7, bilateral HA wearers had higher scores than any other configuration, indicating a better musical QoL.

Active engagement scores for bilateral HA wearers (M = 41.21) were not significantly different from the Gold-MSI norm. However, bilateral CI (M = 24.33) and bimodal (M = 23.62) were both significantly below the Gold-MSI mean (*p* < 0.001). Hearing device type significantly affected perceptual abilities (F = 13.59, *p* < 0.001), creating a strong device hierarchy (HA, bimodal, bilateral CI). Hearing devices had the strongest effect on perception (η^2^ = 0.283) across all Gold-MSI domains. Musical training scores for HA users (M = 27.12) and bimodal users (M = 24.29) approach the Gold-MSI norm (M = 26.52). The device effect on singing ability (singing in tune, harmonising, singing in public) was not significant for HA users who reported higher singing ability than both CI configurations: HA (M = 26.97). Two-way ANOVA found significant effects for device type (F = 7.65, *p* < 0.001) for emotion identification in music. Music sophistication was associated with hearing device type (F = 8.02, *p* < 0.001). HA users (M = 77.94) scored 26.61 points higher than bilateral CI users (M = 51.33, *p* < 0.001 vs. norm) and 20.99 points higher than bimodal users (M = 56.95, *p* < 0.001 vs. norm). Bilateral CI and bimodal users had similar outcomes. HA users scored close to the general population norms after Bonferroni correction and consistently outperformed both bilateral CI and bimodal device users.

### 3.6. LDAs’ Engagement in Musical Activities (H3)

**Figure 8:**
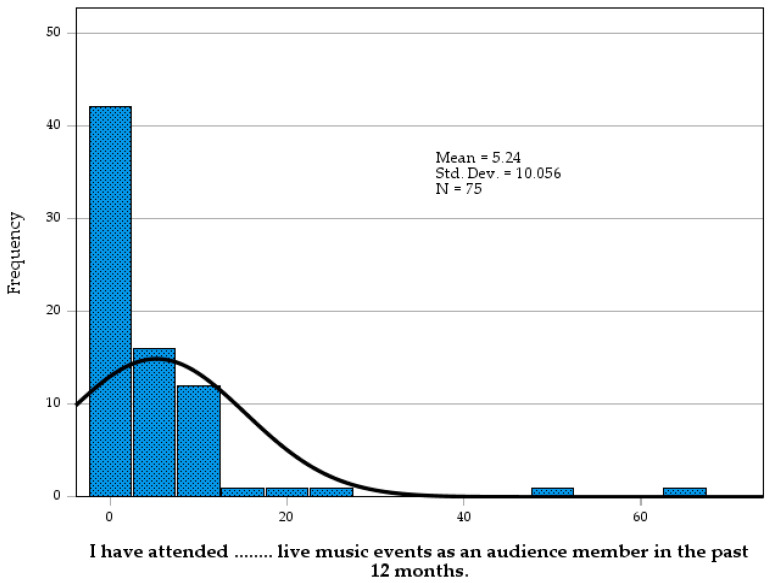
Events attended in the past 12 months, Gold-MSI.

Each of the questionnaires included aspects of participation and active engagement for musical QoL. CIQoL included enjoyment of music and the ability to recognise melodies and hear the clarity of music. Gold-MSI measured active engagement, singing, and the physical and emotional responses music evokes. The MuRQoL questionnaire related the perception and importance of music for such musical aspects as listening, singing, or finding new music. Due to the skewed distribution of event attendance (Figure 8), median values were used rather than mean, which provides a more representative measure, eliminating the confounding factor of outliers. Outcomes from the Gold-MSI questionnaire revealed frequent live music attendance was concentrated among a small selection of LDAs, mostly HA users (n = 18 of 33). When compared to the USA Nielsen Live Music attendance [88], the general population attends two to five events per year. LDA musicians in this study attended between four and six music events. However, non-musicians had dramatically reduced attendance, with a median of only two events for HA users and no CI users attending any events (Table 7).

Hatton [89] (IFPI—International Federation of the Phonographic Industry) reported that the general population listens to music for around 177 min per day per person. This was drastically reduced for LDAs in the current study. Non-musician HA users, despite attending fewer events (Table 7), listened to music for longer (median between 31 and 60 min per day) (Table 8) than musicians across all hearing devices. Daily music listening time was lower for musicians with CI configurations (Table 8), with the median falling in the 0–15 min category compared to HA musicians at 16–30 min per day. All LDA groups, musicians and non-musicians, and hearing device type wearers listened considerably less than the population average of 177 min per day.

## 4. Discussion

The objective of the current study was to capture broad patterns of LDAs’ relationship with music and how HL, hearing devices, and musicianship change music experiences and the subsequent consequences to QoL. As reported in the systematic literature review [46], the focus in HL and hearing device research, coupled with music, focuses on controlled testing of LDAs’ accurate identification of music components (pitch, rhythm, melody, timbre). There are few research studies which assess subjective impacts of HL on music experience for LDAs in relation to either hearing devices or musicianship. Gfeller et al. [13] confirmed this, saying that research studies do not focus on real-life musical and psychosocial experiences for LDAs.

The finding that music enjoyment, rather than music perceptual abilities, was the strongest predictor of QoL has important clinical implications (H1). Even when participants experienced impaired music perception, their QoL still benefited if they derived enjoyment from music. Rehabilitation should not only focus on perceptual abilities but should also facilitate positive emotional experiences with music. When music is not enjoyed, this can exacerbate the negativity of music loss which may be detrimental to QoL. When the sound is poor and not what is remembered with little reward, this can lead to giving up music altogether. This finding validates the difficulty in persevering with music in the hope of improvement.

We found that while the importance of music did not improve perception, it did mediate the mismatch between perception and enjoyment. Analysis confirmed that when participants generally considered music important, they maintained an aspiration for music despite hearing limitations and reduced perception. The findings support a significant positive correlation between the importance and enjoyment of music and that better music perception also led to increased music importance. However, an important finding is that although 73% of participants rated music as always important, only 23% could always enjoy it, and this adversely impacted QoL. This was particularly pronounced for musicians, with 100% considering music important but only 29% always enjoying it. This reinforced findings from previous research confirming that hearing devices present challenges for music enjoyment, resulting in reduced music engagement [13]. However, Akbulut et al. [90] pointed out that for CI users, music’s importance is not necessarily about enjoyment, and our findings reiterate that a love of music remained, even without enjoyment.

We also hypothesised that a relationship exists between perception and music importance, which impacts positively or negatively on QoL (EQ1). When the perception–importance relationship was investigated, a significant relationship was found between music perception and importance, irrespective of musicianship and hearing devices used. Investigation of the perception–importance relationship within subgroups found that musicianship affects both perception and importance, but hearing devices affect perception and not importance. This reveals insights into the differences in a musical relationship between musicians and non-musicians. It is known, from a large body of research [46], that HL and hearing devices generally impact negatively on music perception. Lassaletta et al. [91] reported that people who have more musical training can find music perception sounding mechanical through a CI, whereas non-musicians showed a greater acceptance of technological limitations, focusing more on the experiences of music [92,93]. The findings in the current research extend this understanding, showing that HL or hearing devices do not just result in degraded perception, but they also affect the importance of music. Musicianship is part of identity, so deeply ingrained that musicians do not simply stop being musicians. However, when the quality of music is diminished, their perception, engagement, and the value they place on music are affected. However, for non-musicians, the relationship to music remains relatively stable, suggesting their connection to music is more about appreciation and participation centred around associations, meaning, and connections rather than the technical quality of music.

Previous musical background has been reported as improving musical outcomes [5,89,90,94], but in other studies, musicianship history was not found to improve music outcomes [95,96,97]. Qualitative results [98] of the current study provided greater insight into this apparent polarity, suggesting that previous music training, while it may not improve LDAs’ music perception, does provide resilience and motivation to persevere with the maintenance of musical activities, despite diminished perception. Musicians are driven to engage with music because music is central to their identity, and this may explain why they persevere with music despite degraded fidelity [13]. In this current study, musicians maintained active engagement, which positively influenced musical QoL outcomes regardless of the hearing device worn. Nevertheless, this required persistence with the hope of improvement, and Gfeller et al. [13] found that when there was little reward (i.e., sound was “horrible”), not everyone had the time or tenacity to continue with music engagement.

The MuRQoL questionnaire demonstrated sensitivity to the impacts of musicianship, which was not observed in the general CIQoL. questionnaire When individual MuRQoL domains were compared, the pattern revealed musical QoL expressed through behavioural choices driven by enjoyment rather than simply music perception. Behavioural choices included such things as listening in the car, singing, exploring new music, sharing music, attending live events, creating music, and integrating music into daily life. Ayyildiz et al. [9] pointed out that active engagement with music when alone or in a group is beneficial for QoL. The current research highlighted that active engagement supported musical identity, personality, lifestyle, and improved musical QoL far more powerfully than accurate identification of musical components alone. However, when music was considered important, but it was not enjoyed, it created a paradox: what is it that is important about music when the sensory aspects of music cannot be enjoyed? The survey could not directly answer this question, but qualitative stages provided insights suggesting that a continuing love and importance of music is based on meaning, connections, memories, and personal identity [98], and this has important implications for understanding music’s role in QoL.

The Gold-MSI questionnaire focused on music sophistication, and we expected LDA’s music sophistication (H2) would be lower than the music sophistication of typical hearing people and that this would impact differently for each subgroup. No previous research studies were found which compared HL, hearing devices, and musicianship with music sophistication, and direct comparisons could not be drawn. Participants answered this current survey from the point of view of their hearing status reality, which means their scores captured how they engage with music, given the limitations of their HL or hearing devices. Different patterns emerged between musicians and non-musicians, with non-musicians scoring significantly below the Gold-MSI norms. Musicians scored above the Gold-MSI norms for music training (which was to be expected) but were statistically no different from the general population for other domains, an encouraging finding given their HL. HA users were close to the general population norms, with bilateral CI users showing the lowest scores. This suggests that, regardless of hearing device, musicians maintained near normal levels of musical sophistication compared to non-musicians who experienced significant barriers. The Gold-MSI analysis revealed, despite reduced music fidelity, that LDAs still engaged with music, singing, choosing to listen to music, attending music venues, and playing or performing, which reinforced that music maintained its importance

While there was no interaction between musicians and device types, 77% of musicians had positive QoL outcomes, suggesting that musicianship provided a protective factor regardless of device type. Although we did not directly test this, based on the existing literature that supports musical training being associated with better perceptual abilities [95,96,97,99], we presume their musicianship, training, and experiences better allowed them to extract music from the degraded signal provided by frequency and technological limitations. Lo et al. [95] found speech in noise was improved through both passive and active music engagement, and Hennessy et al. [97] reported musicians performed better than non-musicians. Akbulut et al. [90] stated that for CI users, music significantly impacts positive QoL, while McCrary et al. [94], in a systematic review and meta-analysis, reported that the inclusion of music was associated with clinical improvements in health-related QoL. This has been reported in previous research, which suggested that because LDAs have memories of music, this contextual clue can aid in making sense of music through hearing devices [100].

The final hypothesis (H3) was that LDAs’ participation in musical activities would be less than that of the general population. This hypothesis was strongly supported, with LDAs in this study reporting they did not actively engage in musical activities as frequently as typical hearing people. It is critical to understand how hearing device technology constrains music experiences. HAs cannot restore sound to the dead regions in the cochlea [101]; thus, there are portions of the musical spectrum which are inaudible. Bimodal (HA and CI) users experience timing delays between devices, which may disrupt music continuity, and CI technology is constrained by limited spectro-temporal resolution, which reduces the quality of sound needed for music [102]. While the temporal structure of music can be perceived, spectral resolution, essential for the identification of pitch, timbre, and harmony, is degraded [16,21,102,103,104,105].

These technological constraints have implications for rehabilitation. Even when hearing devices are optimally programmed, they cannot provide the acoustic fidelity listeners want for optimal music enjoyment. Thus, rehabilitation should aim to maximise music engagement, factoring in technological constraints. By identifying subgroups based on musicianship and hearing devices, a clearer picture emerged of different needs.

Musicians, while still reporting reduced active engagement, maintained better engagement than non-musicians. CI users showed greater limitations than HA users. Frequent live music attendance was concentrated among a small selection of LDAs, mostly HA users. Confounding factors, such as the length of HL, may contribute to participants’ disengagement from musical activities. The combination of technology limitations and reduced perception, despite music’s importance, creates a significant barrier to active engagement in musical activities. We know that LDAs socially withdraw [106], and this study provides evidence that this is also true in the musical domain. Withdrawal is associated with social isolation and loneliness, linking to poorer health outcomes, including increased mortality [106]. The implication is that if someone is highly engaged with music, such as attending concerts, playing instruments, singing, or listening, but does not enjoy it, this leads to a lower QoL. But if someone enjoys and is engaged with music, this provides a higher QoL because enjoyment is forefront. This finding provides a compelling explanation for why some LDAs, particularly CI recipients, abandon musical activities altogether. Listening, performing, or attending events can reinforce what they have lost and exacerbate depression, loss of hope, and awaken grief. Counselling which validates the emotional loss but supports continued engagement may sustain musical QoL better than technology optimisation alone.

This paper provides an extensive assessment of how LDAs’ musical QoL was impacted due to HL, hearing devices, and influenced by musicianship. The scores of participants in the Gold-MSI and MuRQoL questionnaires provide compelling evidence that hearing technology plays a significant role in LDAs’ music engagement. While HA users had significantly higher positive MuRQoL outcomes and engaged with music more than either bilateral CI or bimodal users, it must be acknowledged that CI users also had a higher degree of HL, usually for a longer duration. Previous studies indicate that neither a HA nor a CI provides high-fidelity music perception [19]. Thus, the poorer musical outcomes associated with CIs may be due to these confounding factors. The findings in the current study are consistent with previous research [46] that hearing devices present challenges for music enjoyment. Musicians demonstrated unique attributes which positively influenced their QoL. However, the standalone QoL questionnaire failed to measure or account for this. Hence, for individuals who are musically inclined, it is critical that some musically related measures are used to capture their lived experience [90].

In conclusion, participants in this study generally considered music to be important and maintained an aspiration for music despite their hearing limitations. Musicians maintained near normal levels of musical sophistication despite HL, although their enjoyment and active engagement were reduced, and this created a paradox: the importance of music remained, but without enjoyment. LDAs’ persistence to continue with music—despite the disconnect between importance and enjoyment—demonstrates the enduring power of music.

## 5. Implications for Practice

These findings have direct implications for clinical practise and the potential to improve counselling practises that better address the musical needs and expectations of individual LDAs. Rehabilitation for LDAs should extend beyond perception testing to include music-specific activities which address social connections, emotional loss, and identity challenges. Therefore, it is crucial to understand personal and meaningful music experiences, tailoring rehabilitation to both musicianship and hearing devices. We recommend the use of the Gold-MSI and MuRQoL questionnaires in clinics to provide relevant information to audiologists about each LDA musical experience and training, which can guide appropriate rehabilitation strategies.

Hearing device technology impacts not only perception but translates to adjustments in real-world musical activities—a powerful finding for clinical decision-making. The impacts across different hearing devices indicate specific music rehabilitation strategies are required depending upon device configurations. Counselling for realistic expectations and understanding the complex relationship between perception, importance, and enjoyment should cater not just to the limitations of each device configuration but also to the importance of music to individuals as part of their QoL.

As Dr Ian O’Brien [107] said, “You can’t ask a musician to stop playing music.” Hearing healthcare professionals need to acknowledge the desire for music continuity despite HL and hearing devices. Hearing health professionals should receive some level of training in music to understand how the limitations of current technologies can be adapted to give the best solution for each individual. This study highlights the importance and need for further investigation into the QoL and subjective music experiences of LDAs.

## 6. Study Limitations

There were numerous confounding factors which individually or in combination could impact the importance of music, such as degree of musicianship, social connections, memories, level of residual hearing, duration of HL, or technology configurations. All participants were volunteers, with most accessing the survey through online HL, hearing device, and music Facebook groups. This may introduce a self-selection bias, as responses may represent individuals who are more interested in music and/or who need more support. However, this was an appropriate way to reach this hidden population. Since this was a cross-sectional study, causal inferences cannot be drawn between music and QoL. However, the study did reveal associations which require further investigation. While self-identification of musicianship is a fairly common way of assessing musicianship, it could also be a limiting factor, adding ambiguity to the study [86]; thus, the results cannot be generalised to all LDAs. Validation through the six-year rule [86] provides additional support. Future research using stratified random sampling would be needed to confirm generalisability. Participants primarily came from Australia and the USA and are generally conditioned to Western music; therefore, differences and music preferences from different cultures were not included. The survey was long, and research fatigue [108] was a likely contributor to participant attrition. The analysis only included three hearing device configurations and did not include smaller subgroups, such as those who used no devices, unilateral HAs, unilateral CIs, or hybrid CIs. There may be differences in music participation and enjoyment within these groups, but the small sample size would not return meaningful results. Previous musicianship, length of HL, psychological attitude to HL and music, their hearing devices, or other variables could be confounding factors. While statistical analysis suggested participants with HAs had used them for longer than CI users, this was expected because HAs can support most levels of HL, while CIs are usually for severe to profound or complete loss. Regardless of each of the limitations, the findings are meaningful, exploring issues for a population which is under-researched.

## Figures and Tables

**Table 2:** QoL music predictors by hearing device and then musicianship (CIQoL, MuRQoL). This table focuses on predictive relationships, and the tables in Appendix A, Table A5 and Table A6) provide descriptive comparisons.

Group	N	R^2^	Adj R^2^	Sig	Significant Predictors	Key Finding
Musicianship						
Non-Musicians	44	0.172	0.087	*p* = 0.110	None	Very weak
Musicians	31	0.238	0.153	*p* = 0.059	None (music enjoyment *p* = 0.086)	All value music equally, so we cannot identify predictors of QoL
Hearing Device						
Bilateral HA	33	0.499	0.428	*p* < 0.001 *	Music enjoyment (β = 0.519, *p* = 0.002)	Enjoyment strongly predicts QoL
Bimodal (HA + CI)	21	0.602	0.503	*p* = 0.004 *	None individually significant	No clear individual predictor for QoL
Bilateral CI	21	0.332	0.165	*p* = 0.145	None	No musicality attributes predict QoL

* Statistically significant.

**Table 3:** Hearing device impact on music enjoyment when music is always important. Music importance vs. music enjoyment.

**Hearing Device**	**Music is** **Always** **Important**	Music Enjoyment *				
		Always Enjoy n (%)	Enjoy Most of the Time n (%)	Enjoy Sometimes n (%)	Rarely Enjoy n (%)	Never Enjoy n (%)
Bilateral HA (*n* = 33)	28 (85%)	10 (36%)	11 (39%)	6 (21%)	1 (4%)	0 (0%)
Bilateral CI (*n* = 21)	14 (67%)	4 (29%)	4 (29%)	0 (0%)	2 (14%)	4 (29%)
Bimodal (*n* = 21)	13 (62%)	2 (15%)	5 (38%)	2 (15%)	3 (23%)	1 (8%)

* Percentages in enjoyment columns are calculated from the number of participants rating music as Always important for each hearing device.

**Table 4:** Importance of music versus perception of music impact on QoL (n = 75). MuRQoL.

	**Low Perception** Never/Rarely/Occasionally	**High Perception** Frequently/Always
**Low Music Importance** Not important/not very important	Weak-negative QoL impact n = 11 (14%)	Weak-positive QoL impact n = 4 (5%)
**High Music Importance** Somewhat/very/ extremely important	Strong negative QoL impact n = 24 (32%)	Strong positive QoL impact n = 36 (48%)
Green = negative QoL, Blue = positive QoL. Lighter colours indicate weak and darker colours indicate Strong QoL impact		

**Table 5:** Musicians versus non-musicians and hearing device type related to MuRQoL.

	Musicianship			Hearing Devices			
**Influence on QoL**	Musicians	Non- Musicians	TOTAL Musicians	HA	Bilateral CI	Bimodal	TOTAL HD
Strong Negative	7	17	24	2	10	12	24
Weak Negative	0	11	11	3	5	3	11
Weak Positive	1	3	4	2	1	1	4
Strong Positive	23	13	36	26	5	5	36
TOTAL	31	44	75	33	21	21	75

**Table 6:** ANOVA results for MuRQoL perception versus importance of music—musicianship and hearing devices.

MuRQoL Component	Comparison	F	*p*-Value
Perception	Hearing device	8.50	<0.001
	Musicianship	10.48	0.002
	Interaction	0.91	0.408
Importance	Hearing device	0.59	0.557
	Musicianship	22.42	<0.001
	Interaction	2.87	0.063
Overall	Hearing device	4.48	0.015
	Musicianship	25.03	<0.001
	Interaction	2.13	0.127

Note: R^2^ values: perception = 0.356, importance = 0.288, overall = 0.399.

**Table 7:** Median music event attendance, Gold-MSI musicians vs. non-musicians and hearing device.

**Music Events per Year**	**Musicians** (n = 31)		**Non-Musicians** (n = 44)	
	(n)	Median	(n)	Median
Bilateral HA	18	6.5	15	2
Bilateral CI	5	5	16	0
Bimodal	8	4	13	0

**Table 8:** Gold-MSI daily listening (minutes) by musicians, non-musicians, and hearing devices.

**Daily Listening Time Categories (Minutes)**	**Musicians** (n = 31)		**Non-Musicians** (n = 44)	
	(n)	Minutes	(n)	Minutes
Bilateral HA	18	16–30	15	31–60
Bilateral CI	5	0–15	16	0–20
Bimodal	8	0–15	13	0–15

Categories represent the range containing the median daily listening time.

## Data Availability

Data is unavailable due to privacy or ethical restrictions.

## Abbreviations

The following abbreviations are used in this manuscript:

CI/s	Cochlear implant/s—implanted prosthesis of any brand that bypasses the damaged portion of the cochlea. When the external processor is connected, it provides a sense of sound [18,92]
HA/s	Hearing aids
HL	Hearing loss/hearing impairment—any level of loss, one or both ears, caused by any aetiology, treated or untreated
LDA/s	Late-deafened adults, typical hearing people who experienced HL in later life
QoL	Quality of life—including aspects of social wellbeing, emotional and physical health, communication, autonomy, and social integration
CIQoL	Cochlear Implant Quality of Life [51]
Gold-MSI	Goldsmith’s Musical Sophistication Index [55]
MuRQoL	Music-Related Quality of Life [93]

## Appendix A

**Table A1:** Facebook groups where survey was promoted.

Type	Facebook Groups	Approx Membership	Link to Facebook Group
Cochlear Implant	Bilateral CI Warrior	1300	https://www.facebook.com/groups/325256355436065 (accessed on 19 February 2026)
	Cochlear Implant Experiences	46,000	https://www.facebook.com/groups/176991415675866 (accessed on 19 February 2026)
	Cochlear Implant Basics	100	https://www.facebook.com/groups/441587200809972 (accessed on 19 February 2026)
	Cochlear Implants —Australian Support Group	1700	https://www.facebook.com/groups/1554486584854036 (accessed on 19 February 2026)
	Cochlear Implant Users	17,000	https://www.facebook.com/groups/153601038015605 (accessed on 19 February 2026)
	Cochlear Awareness Network	900	https://www.facebook.com/groups/10581738294 (accessed on 19 February 2026)
Hearing Loss	Hearing Loss: The Emotional Side	3000	https://www.facebook.com/groups/1636847240030166 (accessed on 19 February 2026)
	My Hearing Loss Story Group	2100	https://www.facebook.com/groups/1422427341250724 (accessed on 19 February 2026)
	Hearing Aid Forum: Hearing Loss, Hearing Aids, and Cochlear Implants	27,000	https://www.facebook.com/groups/hearingaidforum (accessed on 19 February 2026)
	Australia Hearing Loss Community	1670	https://www.facebook.com/groups/1606706229639531 (accessed on 19 February 2026)
Music, Hearing Loss, Cochlear Implant	Cochlear Implants: How to Enjoy Music—Self Guide to Rehab & Appreciation	650	https://www.facebook.com/groups/MusicAndCIs (accessed on 19 February 2026)
	Association of Adult Musicians with Hearing Loss	1500	https://www.facebook.com/groups/aamhl (accessed on 19 February 2026)
	Musicians with Hearing loss/Tinnitus	200	https://www.facebook.com/groups/musicianswithhearingloss (accessed on 19 February 2026)

**Table A2:** Additional contacts for survey invitation.

	Additional Promotion	Approx Numbers	Link to Web Site
Cochlear Implant	Cochlear Implants South Australia Hearing Services	335	https://www.sacic.com.au/ (accessed on 19 February 2026)
Hearing Loss	Better Hearing Australia	Unknown	https://www.betterhearingaustralia.online (accessed on 19 February 2026)
	Australian Hearing Hub	Unknown	https://hearinghub.edu.au/research-innovation/participate-in-research/ (accessed on 19 February 2026)

**Table A3:** Demographic characteristics by age group, gender, employment, and education (*n* = 75). Values are shown as female (male).

Age Group Category	30–49	50–69	70+	Sub- Total F(M)	Total
Employment status—total Female (male)	11 (0)	18 (12)	18 (15)	47 (27)	74
Retired		0 (3)	2 (1)	2 (4)	6
Unemployed	4 (0)	3 (3)		7 (3)	10
Part-time employment	3 (0)	2 (1)	1 (0)	6 (1)	7
Full-time employment	3 (0)			3 (0)	3
Self-employed	1 (0)	13 (5)	15 (14)	29 (19)	48
Education level—total Female (male)	11 (0)	19 (13)	18 (15)	48 (27)	75
Postgraduate degree	5 (0)	6 (4)	9 (3)	20 (7)	27
Undergraduate degree	4 (0)	9 (5)	9 (2)	22 (7)	29
Technical college	1 (0)	1 (1)	0 (4)	2 (5)	7
Apprenticeship			0 (1)	0 (1)	1
High school	1 (0)	3 (2)	0 (5)	4 (7)	11

Note: Employment status total is 47 (27) = 74 (one missing response). Education total is 48 (27) = 75.

**Table A4:** Duration of hearing device use. Demographic characteristics.

Duration of Device Use *	Mean Years	(n = 71) *
Bilateral HA	13.5	31
Bilateral CI	6.8	20
Bimodal CI right, HA left	CI 4.3/HA 16.3	10
Bimodal CI left, HA right	CI 3.8/HA 12.5	10

* Four participants did not provide duration.

**Table A5:** MuRQoL comparison by hearing device, musicians, and non-musicians.

Hearing Device	Bilateral HA		Bilateral CI		Bimodal		ALL	
MuRQoL	*M*	*SD*	*M*	*SD*	*M*	*SD*	*M*	*SD*
ALL	(*n* = 33)		(*n* = 21)		(*n* = 21)		(*n* = 75)	
Perception	61.76	13.64	40.97	21.24	42.16	19.59	50.45	20.21
Importance	69.83	16.75	60.85	24.03	62.54	18.02	65.27	19.55
Overall	65.80	13.14	50.91	18.89	52.35	15.13	57.86	16.83
Musicians	(*n* = 18)		(*n* = 5)		(*n* = 8)		(*n* = 33)	
Perception	65.11	10.29	51.77	32.01	54.60	19.36	60.25	17.85
Importance	74.10	13.83	87.35	7.25	73.53	13.03	76.09	13.44
Overall	69.60	9.37	69.56	18.05	64.06	10.78	68.17	11.23
Non-Musicians	(*n* = 15)		(*n* = 16)		(*n* = 13)		(*n* = 44)	
Perception	57.75	16.29	37.59	16.66	34.50	15.96	43.55	19.04
Importance	64.71	18.91	52.57	21.19	55.77	17.63	57.65	19.69
Overall	61.23	15.71	45.08	15.38	45.14	12.88	50.60	16.40

**Table A6:** Gold-MSI norm domain outcomes compared to musicians and non-musicians.

	General Population	LDA Musicianship		
Domain	Gold-MSI mean (n = 147,633)	Musicians (n = 31)	Non- musicians (n = 44)	ALL
Active Engagement	41.52	41.81 *p* > 1.000	24.34 *p* < 0.001	31.56 *p* < 0.001
Perceptual Abilities	50.20	46.32 *p* = 0.678	30.59 *p* < 0.001	37.09 *p* < 0.001
Musical Training	26.52	38.13 *p* < 0.001	13.57 *p* < 0.001	23.72 *p* = 0.575
Singing Abilities	31.67	29.71 *p* > 1.000	18.70 *p* < 0.001	23.25 *p* < 0.001
Emotions	34.66	34.94 *p* > 1.000	26.68 *p* < 0.001	30.09 *p* < 0.001
Overall Sophistication	81.58	88.81 *p* = 0.150	47.57 *p* < 0.001	64.61 *p* < 0.001

**Table A7:** Gold-MSI domain norm outcomes compared to HA, bilateral CI, and bimodal users.

	Norms	Hearing Devices (3 Devices × 6 Domains)		
Domain	Gold-MSI Mean (n = 147,633)	HA (n = 33)	Bilateral CI (n = 21)	Bimodal (n = 21)
Active Engagement	41.52	41.21 *p* = 1.000	24.33 *p* < 0.001	23.62 *p* < 0.001
Perceptual Abilities	50.20	46.30 *p* = 0.400	26.86 *p* < 0.001	32.86 *p* < 0.001
Musical Training	26.52	27.12 *p* = 1.000	17.81 *p* = 0.062	24.29 *p* = 1.000
Singing Abilities	31.67	26.97 *p* = 0.098	19.10 *p* < 0.001	21.57 *p* = 0.001
Emotions	34.66	34.85 *p* = 1.000	26.76 *p* < 0.007	25.95 *p* = 0.002
General Sophistication	81.58	77.94 *p* = 1.000	51.33 *p* < 0.001	56.95 *p* < 0.001

Bonferroni-adjusted *p*-values reported (original *p* × 18 for 3 devices × 6 domains).
